# Wiedemann-steiner syndrome with a de novo mutation in *KMT*2*A*: A case report

**DOI:** 10.1097/MD.0000000000019813

**Published:** 2020-04-17

**Authors:** Liu Jinxiu, Liang Shuimei, Xue Ming, Liu CS. Jonathan, Liu Xiangju, Duan Wenyuan

**Affiliations:** a Yinfeng Medical Laboratory, Jinan Shandong; b Genetics Diagnostic Lab, Tai’an Maternity and Child Care Hospital, Tai’an, China; c SoftGenetics LLC, 100 Oakwood Ave, State College, Pennsylvania 16803, USA.

**Keywords:** amblyopia, blepharophimosis-ptosis-epicanthus inversus syndrome, *KMT*2*A*, whole exome sequencing, wiedemann-steiner syndrome

## Abstract

**Rationale:**

Wiedemann-Steiner syndrome (WDSTS, online mendelian inheritance in man 605130) is a rare autosomal dominant disorder characterized by hypertrichosis cubiti. Here, we report a Chinese boy who do not show the characteristic of hypertrichosis cubiti, and was misdiagnosed as blepharophimosis-ptosis-epicanthus inversus syndrome at first. We found a de novo frameshift mutation (p.Glu390Lysfs∗10) in the *KMT2A* gene, which was not reported before. Our study increases the cohort of Chinese WDSTS patients, and expand the WDSTS phenotypic and variation spectrum.

**Patient concerns:**

The patient demonstrated typical craniofacial features of blepharophimosis-ptosis-epicanthus inversus syndrome, including small palpebral fissures, ptosis, telecanthus, and epicanthus inversus, besides he had congenital heart disease (ventricular septal defects), strabismus, hypotonia, amblyopia, delayed speech and language development, delayed psychomotor development, and amblyopia (HP:0000646) which was not reported before.

**Diagnosis:**

*FOXL*2 gene was cloned and sequenced, however, there was no mutation detected in this patient. The result of Chromosomal microarray analysis was normal. The patient was diagnosed as WDSTS by whole exome sequencing.

**Interventions:**

The patient received cardiac surgery, frontalis suspension and regular speech and occupational therapy. He also treated with growth hormone (GH).

**Outcomes:**

The patient's symptoms are improved after cardiac surgery and frontalis suspension, he can express himself well now and had a 10 cm gain in height.

**Lessons:**

As the relationship between genotype and phenotype becomes more and more clear, WES is incredibly powerful tool to diagnose the disease of WDSTS.

## Abstract 2

Supplemental Digital Content is available in the text

## 1 Introduction

Blepharophimosis-ptosis-epicanthus inversus syndrome (BPES, online mendelian inheritance in man [OMIM] 110100) is an extremely rare autosomal dominant disorder, which shows malformation mainly on the eyelids. The prevalence of the syndrome is approximately 1 in 50000.^[1]^ Our patient showed palpebral fissures, ptosis, telecanthus, and epicanthus inversus in facial which similar to BPES, and was misdiagnosed as BPES at first. Mutations in the forkhead box L2 (*FOXL*2) gene cause 2 types of BPES distinguished by the presence (type I) and absence (type II) of premature ovarian failure.^[2]^

Wiedemann-Steiner syndrome (WDSTS, OMIM 605130) is a rare autosomal dominant disorder characterized by hypertrichosis cubiti, associated with short stature; consistent facial features, including long eyelashes, thick or arched eyebrows with a lateral flare, downslanting and vertically narrow palpebral fissures; mild to moderate intellectual disability; behavioral difficulties; and hypertrichosis on the back.^[3]^ It was first described by Wiedemann et al^[4]^ in 1989, and later by Steiner et al in 2000.^[5]^ Since then, more than 20 papers reported WDSTS patients. The phenotypic spectrum of the syndrome was widely expanded.

Mutations in the *KMT*2*A* gene have been reported as being responsible for WDSTS.^[6]^ Since then, the genomic spectrum of this syndrome continues to expand.^[7]^ Here, we describe a patient with the typical features of BPES, however a de novo heterozygous truncating variant was identified in the *KMT*2*A* gene after a whole exome sequencing process, and the patient was diagnosed as WDSTS molecularly.

## 2 Case report

**Figure 1:**
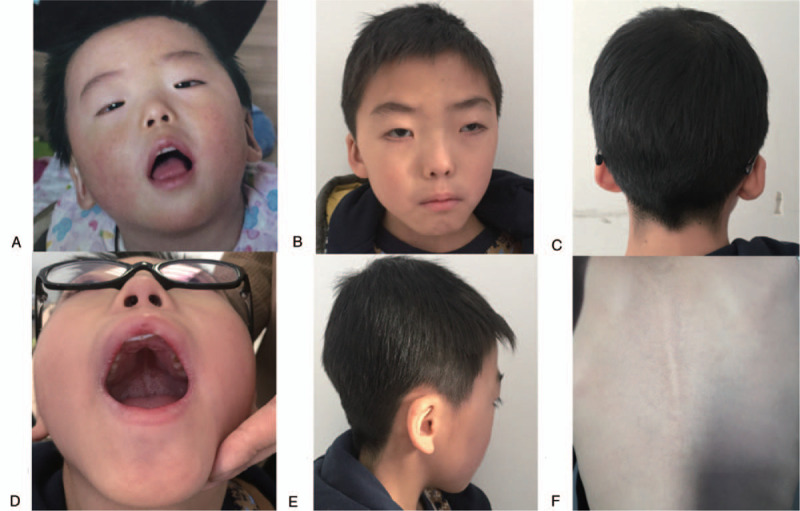
The clinical features of the patient. (A) The patient at the age of 1 yr, the patient experience a combination of congenital eyelid anomalies: small palpebral fissures, ptosis, telecanthus, and epicanthus inversus. (B) The patient at the age of 7 yr after frontalis suspension, shows thick eyebrows, hair and long philtrum, trabismus and amblyopia; (C) low hairline; (D) narrow high palate; (E) external ear deformity and low-set ears; (F) hypertrichosis back.

The Chinese boy was 10 years old now, he was born at 36 weeks’ gestation as the first child of healthy non-consanguineous Chinese parents. Pregnancy and delivery were normal. At birth, he exhibited weight of 2350 g and length of 45 cm, and hypotonia was noted. Soon after birth, congenital heart disease (ventricular septal defects) was detected, and received operation at 7 months of age. He could walk independently at 2 years old, and his overall developmental quotient was 45 in Gesell development schedule at 3 and a half years old. The patient demonstrated typical craniofacial features of BPES, including small palpebral fissures, ptosis, telecanthus, and epicanthus inversus, as shown in Fig. 1A, in order to see clearly, he raised his head when he watching, and performed frontalis suspension at 4 years old. His eyes were strabismus (Fig. 1B) similar to Yongguo Yu's report.^[8]^ Besides, he had amblyopia (HP:0000646), which was not reported before. Our patient had a bulbous nose, a wide and depressed nasal bridge, thick eyebrows and hair, long philtrum, low hairline, downturned corners of the mouth (Fig. 1B and C) and hypertrichosis back without hairy elbows, external ear deformity, low-set ears. (Fig. 1E and F) and a narrow high palate. (Fig. 1D) He also had early development delay in speech and psychomotor development.

**Figure 2:**
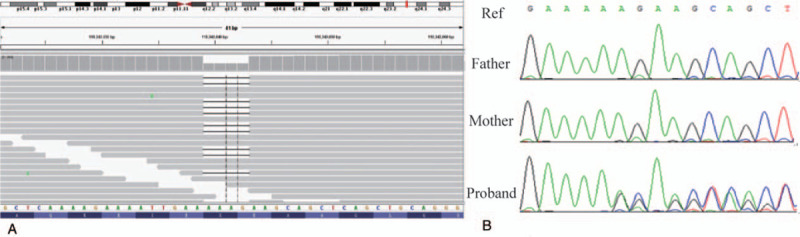
Analysis of possibly causative genetic mutation in the patient. (A) View of aligned sequence reads spanning the *KMT*2*A* variants in the patient using the Integrated Genomics Viewer. (B) Sanger sequencing confirmed a c.1167-1170delAGAA mutation in *KMT*2*A* gene.

Our patient was diagnosed as BPES syndrome initially, according to the clinical phenotype. However, no mutation in *FOXL*2 gene was detected using Sanger sequencing. chromosomal microarray analysis analyses revealed no significant deletion or duplication reported. No more than 10Mb of homozygosity region was found in the patient. One de novo variant was identified by WES: a heterozygous c.1167–1170delAGAA (p.Glu390Lysfs∗10) variant in the *KMT*2*A* gene (NM_001197104.1). WES result for the patient is suggestive of a molecular diagnosis of Widemann-Steiner Syndrome. This variant sits within exon 3 of the *KMT*2*A* gene on chromosome 11 at 118343041 bp which is predicted to result in premature termination of the protein product. Alignment view of this variant in the Integrative Genomics Viewer can be seen in Fig. 2. This variant has been added to *KMT*2*A* variant database in Leiden Open Variant Database, and the DB-ID was KMT2A_000130. Sanger sequencing to the parents demonstrated that the variant is a de novo mutation. It is not present in the 1000 Genomes Project, ExAC, GnomAD, EVS and CNGMD (Ten-thousand people) in-house database, and was not reported in literatures. The mutation could be classified as “pathogenic” according to The American College of Medical Genetics and Genomics variant interpretation guideline.^[9]^ There were no pathological mutation found in *GALC* and *CACNA*1*F* gene which are associated with amblyopia.

The patient's symptoms are improved after cardiac surgery and frontalis suspension. After genetic diagnosis of WDSTS, he received growth hormone therapy (0.15 IU/kg/d = 0.05 mg/kg/d) for 12 months and had a 10 cm gain in height (0.83 cm/month). He also enrolled in special education classes and receives regular speech and occupational therapy, and he can express himself well now.

The study protocol was approved by Ethical review board of Yin Feng Academy of Life Science Table S1.

## 3 Discussion

It has been noticed that WDSTS resembles BPES especially in eyelid features, including small palpebral fissures, ptosis, telecanthus, and epicanthus inversus et al. WDSTS is easily misdiagnosed as BPES syndrome when the patient do not have hairy elbows. Up to now, more than 83 WDSTS patients with variations in *KMT*2*A* gene were confirmed, most of them shared similar clinical features, such as hypertrichosis cubiti or back, developmental delay, cognitive disability, pre and postnatal growth retardation, and a distinctive facial appearance: palpebral fissures, down-slanted, wide nasal bridge, broad nasal tip, long eyelashes, and thick eyebrows. Niu Li et al’ study and our report confirmed that: absent palmar proximal transverse creases is not a unique feature of Chinese patients.^[10]^ Table S2.

Jones et al revealed that heterozygous variations of the *KMT*2*A* gene (OMIM #159555) was responsible for WDSTS in 5 patients in 2012.^[6]^ Since then, more than 20 papers report new mutations about the *KMT*2*A* gene. The Clinvar database (2019/01/25) has reported 96 mutations and small indels classified as Pathogenic, 43 of them are frameshift small indels, 39 are missense mutations and 4 are splicing mutations, nearly 60% of the mutations located in exon 3 and 27, because these 2 exons are much larger than other exons. The *KMT*2*A* gene, also known as *MLL*, encodes a histone methyltransferase that plays a critical role in regulating gene expression during early development. Because *KMT*2*A* regulates multiple Hox and Wnt related genes through histone H3 lysine 4 (H3K4) methylation,^[11]^ phenotypes of the WDSTS patients are complex and involve multiple systems, including craniofacial features, skeletal anomalies,^[12]^ organic problems, development and intellectual disability.^[13,14]^

After genetic diagnosis of WDSTS, necessary genetic counseling should be offered to the patients. Patients should be enrolled in special education classes and receives regular speech and occupational therapy if they showed language delay and intellectual disability. Carntine supplementation was given to the patients if they are hypotonia. The corresponding surgical treatment should be implemented if the patients have cardiac anomaly. In this case, the patient had a good prognosis after receiving cardiac surgery. Most of WDSTS patients characterized developmental delay, Investigation of pituitary function should be undertaken in children with WDSTS. A pituitary MR scan should be considered if there is biochemical evidence of growth hormone deficiency. Recombinant human growth hormone treatment should be considered for treatment of WDSTS with growth hormone deficiency.^[15]^ All these measures enable WDSTS patients to survive better.

As the relationship between genotype and phenotype becomes more and more clear, WES is incredibly powerful tool at identifying disease-causing variants. Vandana Shashi's detailed analyses suggest that next-generation sequencing should be applied when the traditional approaches do not result in a diagnosis after the first visit. Genetic disorders experience long-term psychosocial and economic burden, they are benefit from WES not only in a higher rate of genetic diagnosis but a considerable cost savings.^[16]^ As more patients are diagnosed, the prevalence of WDSTS will be updated.

In summary, we report a boy with a novel *KMT*2*A* mutation from Chinese origin. He do not show 1 of the characteristic WDSTS phenotype, hypertrichosis cubiti, and he also do not had absent palmar proximal transverse crease, which reported by Yu Sun et al.^[17]^ Instead, he had amblyopia, which was not linked to WDSTS patients previously. Our finding extended the WDSTS genetic and phenotypic spectrum.

## Acknowledgments

This work was supported by the grants from the National Basic Research Program of China (2013CB945402, 2013CB945403) and Shandong Provincial Natural Science Foundation (ZR2015CL033). The authors declare there is no conflict of interest. We thank the patient and their family members for their participation.

## Author contributions

**Conceptualization:** Xiangju Liu, Wenyuan Duan.

**Data curation:** CS Jonathan Liu.

**Formal analysis:** Ming Xue.

**Investigation:** Xiangju Liu.

**Methodology:** Jinxiu Liu.

**Project administration:** Duan wenyuan.

**Resources:** Ming Xue, Xiangju Liu.

**Software:** Liu CS Jonathan.

**Supervision:** Wenyuan Duan.

**Writing – original draft:** Liu Jinxiu.

**Writing – review and editing:** Jinxiu Liu, Shuimei Liang.

## Supplementary Material

Supplemental Digital Content

## Supplementary Material

Supplemental Digital Content
