# Supplementary material for: Wiedemann-steiner syndrome with a de novo mutation in KMT2A: A case report
Source: Medicine (Baltimore). 2020 Apr 17;99(16):e19813. doi: 10.1097/MD.0000000000019813 (PMC7440326; doi:10.1097/MD.0000000000019813)
Supplement: Supplemental Digital Content [file medi-99-e19813-s001.doc]

**Table S1 Detailed primer sequence for *FOXL*2 and *KMT*2*A* gene**

| Primer | Primer sequence |
| --- | --- |
| FOXL2-1-F | AGTTTGAGACTTGGCCGTAAGCG |
| FOXL2-1-R | CCATCTGGCAGGAGGCATAGG |
| FOXL2-2-F | GACCCGGCCTGCGAAGACAT |
| FOXL2-2-R | GAGGGGACAAAGAGGAGCGACA |
| FOXL2-3-F | GCGGACTCGTGCGCCCCAACT |
| FOXL2-3-R | AGGCGGGCCCAGAGGGTGTGA |
| KMT2A-Exon3-F | GTTTCAGCAAGCCACAAAGATT |
| KMT2A-Exon3-R | TGAACTTCAGGGGTATCGCTC |
