# Supplementary material for: Wiedemann-steiner syndrome with a de novo mutation in KMT2A: A case report
Source: Medicine (Baltimore). 2020 Apr 17;99(16):e19813. doi: 10.1097/MD.0000000000019813 (PMC7440326; doi:10.1097/MD.0000000000019813)
Supplement: Supplemental Digital Content [file medi-99-e19813-s002.docx]

**Table S2 More information about the whole exome sequencing**

| Sample Name | Good reads | Mapped Reads | Mapped Ratio(%) | Unique Mapped Reads | Unique Mapped Ratio(%) |
| --- | --- | --- | --- | --- | --- |
| Patient | 93935600 | 93773683 | 99.83 | 84009303 | 89.43 |
| Father | 132299638 | 132099208 | 99.85 | 116956573 | 88.4 |
| Mother | 111349326 | 111214053 | 99.88 | 98870357 | 88.79 |


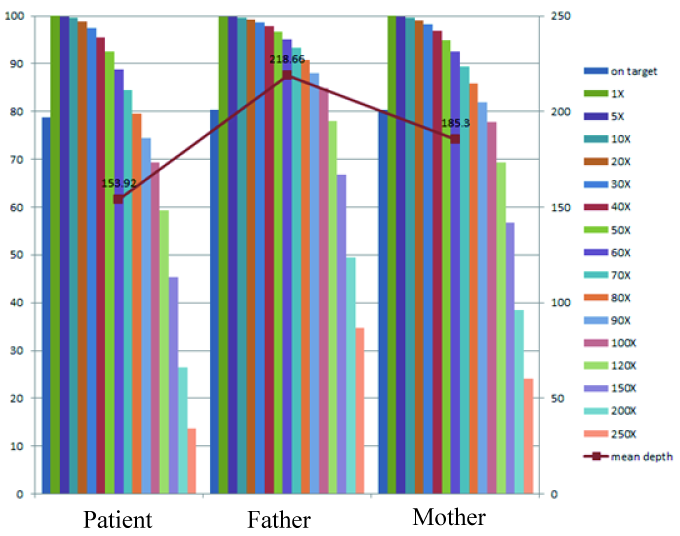


capture efficiency and sequencing depth
